# Case Report: Ventriculoperitoneal shunt for management of syringomyelia and Chiari-like malformation in a Cavalier King Charles Spaniel

**DOI:** 10.3389/fvets.2026.1946635

**Published:** 2026-09-30

**Authors:** Ana Fernandez Cid, Joana Tabanez, Carina Black, Clare Rusbridge

**Affiliations:** 1 Southern Counties Veterinary Specialists, Ringwood, United Kingdom; 2 Lumbry Park Veterinary Specialists, Alton, United Kingdom; 3 Fitzpatrick Referrals, Eashing, United Kingdom; 4 School of Veterinary Medicine, Guildford, United Kingdom

**Keywords:** Chiari-like malformation, syringomyelia, ventriculomegaly, ventriculoperitoneal (VP) shunt, Cavalier King Charles Spaniel

## Abstract

A 1-year-old male Cavalier King Charles Spaniel (CKCS) was presented with spinal hyperesthesia, reluctance to exercise, and phantom scratching. MRI revealed a Chiari-like malformation (CM) with brachycephaly, cerebellar herniation, ventriculomegaly, and syringomyelia extending from the odontoid peg to L5, with a maximum transverse syrinx width of 7.0 mm at C2–C3. Medical management (meloxicam and gabapentin) failed to resolve clinical signs. Due to significantly increased lateral ventricles and concern for syrinx progression, a ventriculoperitoneal shunt was placed. Aside from transient post-operative pneumocephalus, the procedure was successful. The dog showed significant clinical improvement, and follow-up MRI demonstrated syrinx reduction and resolution of lateral ventricles. At 6 months post-op, the syrinx measured 2.3 mm. Eight months after the surgery, the dog experienced a brief episode of a suspected movement disorder like but recovered fully. The patient remains pain-free and neurologically normal. This case describes the clinical and imaging outcomes of ventriculoperitoneal shunting (VPS) in a dog with Chiari-like malformation, syringomyelia, and ventriculomegaly.

## 1 Introduction

Canine Chiari-like malformation (CM) is a complex inherited condition of the skull and craniocervical junction commonly encountered in brachycephalic toy breeds. The resulting overcrowding of the brain and cervical spinal cord leads to pain and/or secondary syringomyelia (SM) (1). Disruption of the cerebrospinal fluid (CSF) circulation caused by neuroparenchyma overcrowding, particularly at the level of the foramen magnum, leads to the formation of fluid-filled cavities within the spinal cord, although the exact pathogenesis of syrinx formation is not fully understood (1–3). Ventriculomegaly, typically of all four ventricles, may also manifest in brachycephalic dogs with CM due to the obstruction of the lateral aperture of the fourth ventricle and possibly due to reduced CSF absorption via the olfactory and skull base lymphatic route (4).

Cavalier King Charles Spaniels (CKCS) and their crosses are commonly affected by CM, demonstrating a high prevalence of at least one craniocervical malformation on a magnetic resonance image (MRI) scan (5). Studies in Europe and the US have suggested that prevalence is approximately 47% across all age groups, with a 1.3 increase in odds ratio per year, showing that prevalence increases with age and 30% of CKCS without syringomyelia would develop it annually (6–9). By 12 months of age, approximately 25% of CKCS show SM, and this prevalence increases substantially (70%) by 72 months or more (10). The most common signs associated with CM are behavioral or caregiver-reported clinical signs suggesting pain, such as vocalization described as either spontaneous or occurring during Valsalva mimics such as jumping, being lifted, or during defecation. Other common clinical signs include spinal pain at any level, reduction in activity, reluctance to jump or climb stairs, sleep disturbance, scratching or rubbing of the head or ears, and personality changes (anxiety, aggression, or withdrawal). In the CKCS, which tends to form syringes in the dorsal gray column of the mid-cervical spinal cord, phantom scratching is one of the most common associated clinical signs (1). Other clinical signs associated with SM include weakness, thoracic limb muscle atrophy, and postural deficits (1).

In humans, Chiari-like malformation Types 0 and I (CM-1) share similarities with CM in dogs, and approximately 60–85% of humans with CM-1 also suffer from SM (11, 12).

Medical management of CM/SM, in both humans and dogs, is based on analgesia, CSF reduction drugs, physiotherapy, hydrotherapy, and acupuncture (1, 13, 14). Surgery is usually reserved for individual cases that fail medical management or have progressive syrinx formation (15). Foramen magnum decompression (FMD) is the preferred surgical technique for CM-I humans, except in the presence of ventriculomegaly. However, 8–30% of humans require reoperation due to the development of scar tissue formation (16).

Other surgical techniques have been described to manage syringomyelia, such as syringo-subarachnoid and syringo-pleural shunting procedures. Additionally, ventriculoperitoneal shunting may be considered in case of ventriculomegaly or hydrocephalus, potentially offering more effective results in achieving syrinx collapse (3, 17–19).

This study aimed to describe the treatment rationale and outcome of a ventriculoperitoneal shunt technique to treat a juvenile CKCS with SM, CM, and ventriculomegaly.

## 2 Case presentation

We present the case of a 1-year-old male entire CKCS that was referred to the neurology department for the assessment of spinal pain. Historical signs of suspected cervical or spinal pain were reported, including lethargy, reluctance to jump, sporadic vocalization, reluctance to eat from a bowl placed on the floor, and phantom scratching. Initial medical management with gabapentin (~6.5 mg/kg/12 h PO, as higher doses were associated with sedation in this patient) and meloxicam (0.1 mg/kg/24 h PO) was prescribed by the primary veterinarian. However, clinical signs of pain continued despite medication. Neurological examination by a European Board-certified neurologist revealed cervical hyperesthesia alone.

MRIs of the brain and whole spine were acquired under general anesthesia using a 1.5T scanner (Tim system, Siemens). The brain protocol included T2-weighted (T2W), T1-weighted (T1W), fluid-attenuated inversion recovery (FLAIR), and T2-weighted gradient echo (T2W GRE). Images were acquired in transverse, dorsal, and sagittal planes as appropriate. The spinal protocol included T2W and T1W images in sagittal and transverse planes, short tau inversion recovery (STIR) images in the dorsal plane, and a three-dimensional constructive interference in steady state (3D-CISS) sequence in the transverse plane (1). With the help of the multiplanar reconstruction software integrated into the MRI scanner, 3D-CISS data were further processed in sagittal and dorsal planes. It revealed a Chiari-like malformation typical for the breed with a significant cerebellar vermis herniation/compression against the atlanto-occipital ligament and laminae of C1 (Figure 1A). There were moderate dilatation of the entire ventricular system, reduced volume of olfactory bulbs, and flattening and ventral rotation of the forebrain (significant brachycephaly conformation). Syringomyelia was reported from the level of the odontoid peg to the L5 vertebra with a patchy central canal dilation more caudally. There was a signal void in the syrinx, suggesting high-velocity fluid, and in the thoracic and lumbar regions, the signal intensity suggested a mix of pre-syringomyelia and syringomyelia. These features were considered as a risk for syrinx expansion and holocord syringomyelia (Figure 2A). The spinal cord segment with the widest syrinx was located approximately C2–C3. The syrinx was symmetrical, with a maximum transverse diameter of 7.0 mm and a cross-sectional area of 35.96 mm^2^.

**Figure 1:**
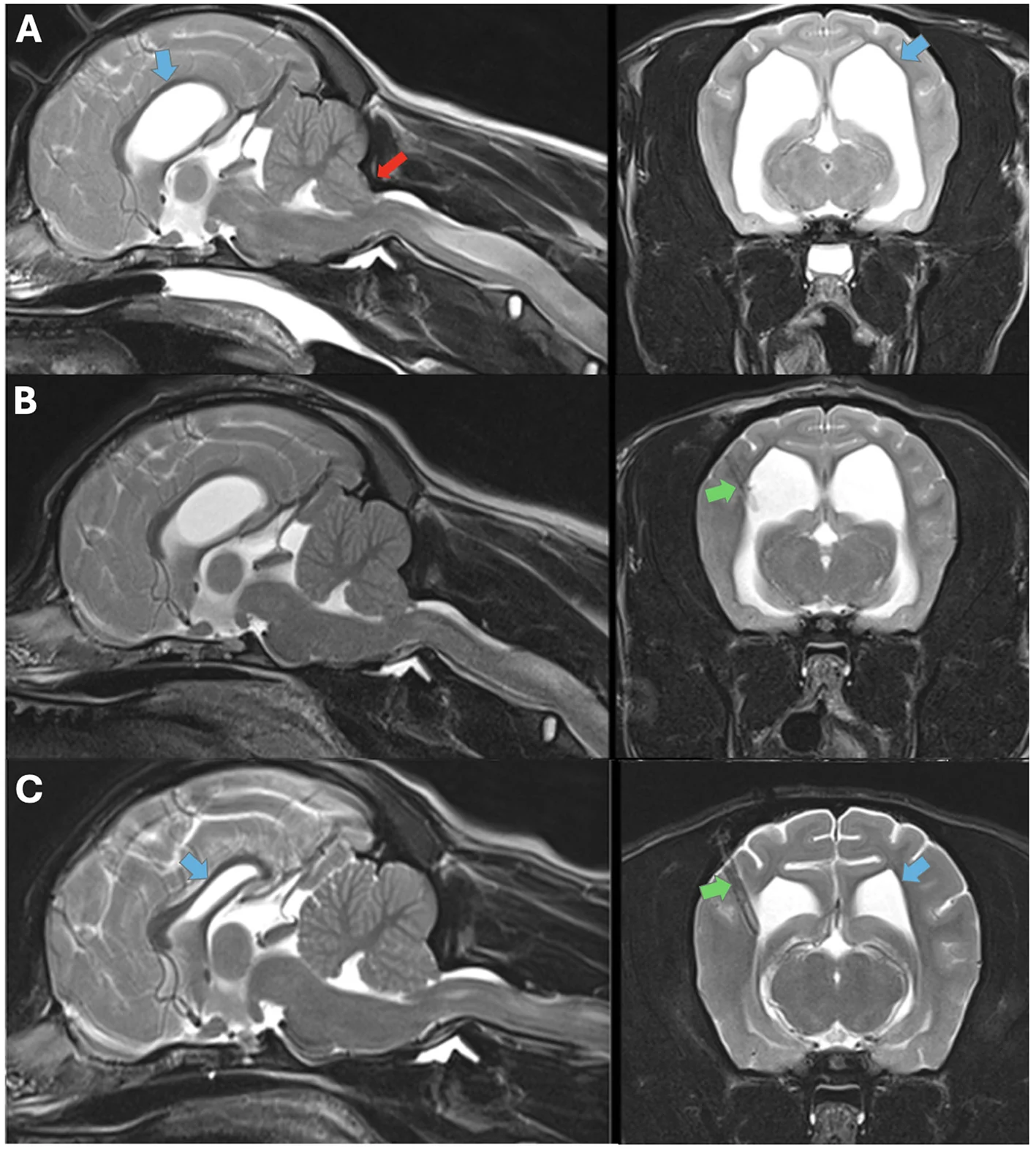
Sagittal and transverse T2-weighted images of the brain pre-operatively **(A)** and at 2 months **(B)** and 6 months post-operatively **(C)**. The ventricular shunt is visible entering the right lateral ventricle [green arrows in **(B, C)**]. Note the initial diffuse ventriculomegaly (blue arrows) and overcrowding of the caudal fossa with rostral flattening of the cerebellum and extension of the caudoventral cerebellar vermis across the foramen magnum [red arrow; **(A)**]. At 6 months post-operatively, marked improvement of the lateral ventriculomegaly was noted (blue arrows), but the third and fourth ventricles displayed similar sizes. The overcrowding of the caudal fossa was also improved with the cerebellum no longer compressed rostrally, and only mild indentation of the caudoventral vermis was noted across the foramen magnum **(C)**.

**Figure 2:**
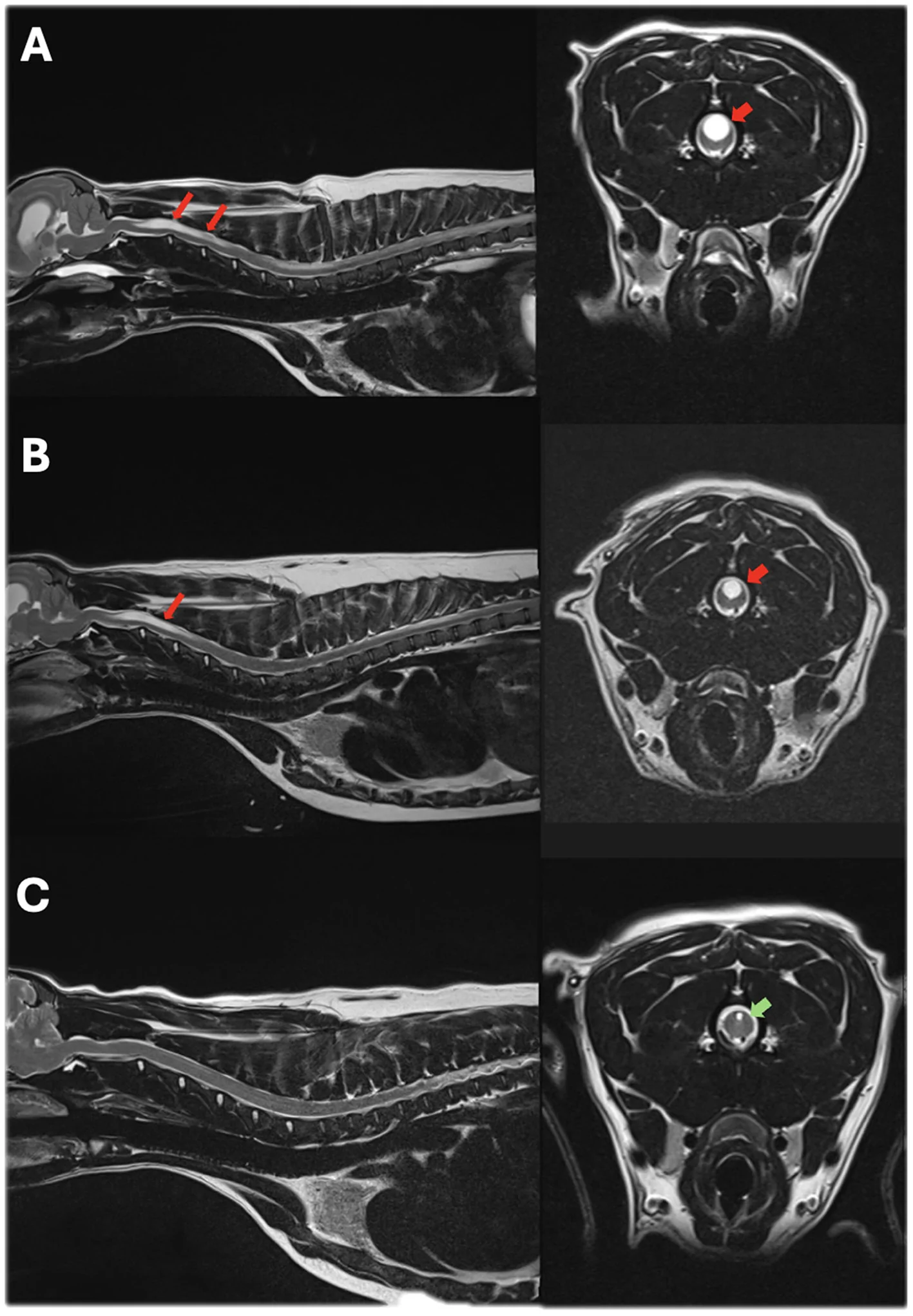
Sagittal and transverse T2-weighted images of the cervical spine pre-operatively **(A)**, at 2 months **(B)**, and 6 months post-operatively **(C)**. The syringomyelia is visible as a well-defined, ovoid-shaped, T2W hyperintense (CSF-isointense) lesion located within the dorsal aspect of the spinal cord and confluent with a distended central canal (*red arrows*). Pre-operatively, the syringomyelia occupied approximately 60% of the vertebral canal cross-sectional area when measured on the transverse images **(A)**. At 2 months post-surgery, it reduced in size, occupying approximately the 45% of the vertebral canal cross-sectional area **(B)**. At 6 months, there was a notable improvement with only mild distension of the central canal observed along the caudal third of C2 the vertebra [*green arrow*; **(C)**].

A cranial approach was performed through incision of the skin, subcutaneous tissue, and temporalis fascia, followed by retraction of the temporal muscle. A craniotomy of the right parietal bone was performed with a 3-mm burr for placement of the ventricular catheter. A second smaller burr hole was performed for the insertion of a non-absorbable monofilament suture to secure the catheter to the skull. The depth of the shunt was measured pre-operatively, and after incision of the dura, the catheter shunt was placed and connected to a high-opening pressure valve (110–170 mm H20) (Medtronic^®^) (Medtronic plc, Dublin, Ireland). Valve selection was justified based on previous similar cases that had post-operative complications of hygroma following a procedure with a medium opening pressure value. This valve was positioned subcutaneously and dorsal to the base of the neck. A third incision was performed on the right flank, 3 cm caudal to the last rib and halfway between the lumbar spine and ventral abdomen, for peritoneal placement of the barium-impregnated distal shunt (Medtronic^®^). Using a long Doyen intestinal forceps, a subcutaneous tunnel was created to connect the abdominal shunt to the neck valve. Computed tomography (CT) of the head and neck was performed under general anesthesia to evaluate the correct shunt placement (Aquilion PRIME, Toshiba Medical System Corporation, Otawara-shi, Tochigi, Japan; 160-slice multi-slice CT scanner), which revealed that the ventricular shunt was slightly too ventral as initially intended, which led to subsequent intervention to retract slightly the length of the shunt. Readjustment of the shunt caused a mild bilateral intraventricular pneumocephalus visible on the post-operative CT scan.

The dog was discharged after 3 days with subtle paraparesis and an equivocal menace response deficit in the right eye. These mild neurological signs were attributed to the surgical approach and presence of pneumocephalus affecting the sensory-motor cortex. Cage rest or small room confinement was advised for the following 2 weeks alongside physiotherapy exercises to be performed at home by the caregivers. Stairs, running, and rough playing were not permitted. The patient was discharged with paracetamol 10 mg/kg/12 h for 10 days, gabapentin 6.25 mg/kg/8 h, and prednisolone 0.6 mg/kg/24 h for 7 days, followed by 0.25 mg/kg/24 h for 7 days, and then discontinued. Gabapentin was administered at a lower dose due to mild sedation observed in the patient. Prophylactic measures following the surgery included the prescription of anti-seizure medication. Levetiracetam was initiated at a dosage of 23 mg/kg/8 h for a duration of 2 weeks. Omeprazole was prescribed at a dosage of 1.2 mg/kg/12 h for 7 days theoretically to reduce CSF production. Additionally, prophylactic antibiotic therapy was administered in the form of cefalexin at a dosage of 25 mg/kg/12 h for 7 days.

## 3 Follow-up and outcomes

The patient was reassessed 2 weeks post-surgery and was neurologically normal without signs of cervical hyperesthesia. At this examination, the dog received only gabapentin at a dosage of 6.25 mg/kg/8 h. A small subcutaneous seroma of the right side of the abdomen was identified.

Six weeks post-operatively, the patient was reexamined and was neurologically normal. Vocalization and reluctance to jump were persistent at 2 weeks post-operatively but resolved thereafter according to the caregivers. Gabapentin was further reduced to twice daily.

At 7 weeks, a repeat MRI was performed, showing the most significant change being a marked reduction in syrinx size on the caudal cervical and cranial thoracic spine. At the level of C2, the larger syrinx diameter was 4.9 mm (Figure 2B). The syrinx was also smaller in the thoracic and lumbar spines. The lateral ventricles were reduced in size (Figure 1B).

A follow-up appointment was scheduled 6 months afterward. The neurological examination remained normal, and phantom scratching could not be elicited. The caregiver reported a few sporadic yelping episodes without a specific pattern or frequency. The dog continued to receive a low-dose gabapentin at a dosage of 6.25 mg/kg/12 h. Another MRI in the cervical region demonstrated further syrinx collapse with a transverse width of 2.3 mm and a cross-sectional area of 2.28 mm^2^, indicating almost complete resolution of the syringomyelia (Figure 2C). Lateral ventriculomegaly had also improved with the third and the fourth ventricle dilatation remaining similar (Figure 1C).

The ventriculoperitoneal shunt remained in its original position. Gabapentin was discontinued, and the dog's caregiver did not report further clinical signs. Until 37 weeks post-operatively, the caregivers reported an acute onset of a movement disorder-like episode that lasted approximately 60 min. The episode occurred after rough play with another dog and was characterized by generalized incoordination, disorientation/lethargy, and a swaying gait that lasted approximately 5 min. No head tilt or nystagmus was observed. The episode resolved spontaneously and completely. By the time of the veterinary examination, the patient was neurologically normal with no spinal hyperesthesia. The etiology was unknown, but given the short duration and complete resolution, a shunt-related issue was considered less likely.

We contacted the caregiver 2 years after diagnosis, and the dog remained free of clinical signs and received medication. Phantom scratching resolved. No further movement disorder-like episodes were reported.

## 4 Discussion

The present case describes a 1-year-old male Cavalier King Charles Spaniel with clinical signs of cervical pain and phantom scratching, later diagnosed with CM-SM, a condition commonly associated with this breed (2–4). Despite initial medical management with gabapentin and meloxicam, clinical signs persisted, necessitating further diagnostic imaging. MRI findings revealed CM, ventriculomegaly, and wide SM with evidence of progressive disease (5). Surgical intervention with ventriculoperitoneal shunt placement resulted in significant clinical improvement, as demonstrated by reduced syrinx size and resolution of clinical signs in follow-up assessments and up to the date. Notably, the patient's neurological function normalized by 6 weeks post-operatively, with MRI confirming near-complete resolution of the SM and remaining neurologically normal 2 years after. This case highlights the efficacy of early surgical intervention in managing CM/SM in affected breeds while also emphasizing the need for careful post-operative monitoring, imaging, and adjustment of treatment plans based on individual patient response.

The therapeutic approach to CM/SM in both human and canine patients primarily focuses on pain relief, medications to reduce CSF accumulation, and non-invasive treatments such as physiotherapy, hydrotherapy, and acupuncture (6–8).

In a study of CKCS medically managed for CM/SM over 39 months, the authors found that 75% of the cases had progression of clinical signs (9). Surgery is generally reserved for cases that do not respond well to medical interventions or for those with worsening SM (19).

Ventriculomegaly in dogs with Chiari-like malformation (CM) may, at least in part, exhibit altered cerebrospinal fluid (CSF) dynamics and impaired outflow from the ventricular system. In dogs, the ventricular system communicates with the subarachnoid space primarily through the paired lateral apertures of the fourth ventricle, as a median aperture is absent. The cranial and craniocervical conformational abnormalities associated with CM, including overcrowding of the caudal cranial fossa, may compromise CSF flow through this region and through the lateral apertures. Consequently, impaired ventricular CSF outflow could contribute to ventricular dilatation and may provide a pathophysiological rationale for CSF-diverting procedures, such as ventriculoperitoneal shunting, in selected affected dogs (10).

In humans with CM-I, the preferred surgical option is foramen magnum decompression (FMD), except when ventriculomegaly is present; a CSF diversion procedure is usually indicated. In humans, reoperation is necessary in 8–30% of cases due to the formation of significant scar tissue at the surgical site (20). In an attempt to address this complication, an American clinic introduced FMD with cranioplasty, a technique that has since been adapted for veterinary use in dogs (11, 12). However, this technique has never been evaluated in large groups with short (only immediate post-operative MRI) or long-term post-operative MRI. FMD in dogs with CM/SM is reported to significantly reduce pain-related signs. In a case series, approximately 80% of CKCS demonstrated favorable outcomes following FMD without cranioplasty. In these surgical cases, syrinx resolution was not achieved, contrary to our case. Consequently, a significant proportion of dogs required long-term pain management with medications such as gabapentin, pregabalin, or intermittent NSAIDs to address neuropathic pain syndromes (9, 13).

In two other reports comprising a total of 36 dogs (21 in one study and 15 in the other), 25–47% of those undergoing FMD without subsequent cranioplasty required revision surgery, and post-operative MRI evaluations showed no significant changes in the syrinx among those imaged (16).Notably, this percentage may underestimate the true revision rate, since some caregivers may have declined additional surgery. A different case series described eight dogs undergoing FMD with occipital cranioplasty with customized titanium prostheses for CM. Of the five dogs that had post-operative MRIs at 6–20 months, only three showed a 30–50% decrease in syrinx size, and five required long-term medication (11).

CSF diversion procedures, such as ventriculo-, syringo-, or subarachnoid-to-subarachnoid or peritoneal shunts, are indicated in humans who fail to respond to FMD, have isolated syringomyelia, or present with significant ventriculomegaly or hydrocephalus on imaging (14, 16). It is under one of the author's impressions, supported by computer modeling, that in many dogs, a syrinx develops relatively quickly and then remains largely unchanged, reaching a dynamic equilibrium (5, 6). Indeed, previous canine FMD case series do not describe reversal of the filling mechanism or substantial shrinkage of the syrinx, suggesting that CSF diversion procedures are a better option (13). In the present case, a ventriculoperitoneal shunt was chosen for this reason and due to the presence of ventriculomegaly, offering improvements in syrinx collapse.

In the present case, ventriculoperitoneal shunt was selected because of the concurrent ventriculomegaly and the suspected contribution of altered ventricular CSF dynamics to syrinx formation and maintenance. Diversion of CSF from the ventricular system was therefore considered a logical approach to modify the pressure gradients contributing to ventricular dilatation and syrinx persistence while also offering the potential for syrinx collapse.

A separate study involving 11 dogs (CKCS and Yorkshire Terriers) reported surgical management of CM-SM via syringo-subarachnoid shunts placed in either the cervical or lumbar region. Although the majority of patients demonstrated neurological improvement, the absence of post-operative MRI imaging limits the ability to objectively assess the extent of anatomical or pathological resolution. Nearly all were managed long-term on furosemide (17). A similar case report described a CKCS treated with a syringo-subarachnoid shunt, which resulted in markedly reduced neuropathic pain, although intermittent episodes still occurred (18). In contrast, this study included comprehensive post-operative MRI and noted no clinical signs without long-term medication. Finally, a syringo-pleural shunt was successfully used in a Pug dog with a cervical diverticulum and syringomyelia, though it was not associated with CM. While the diverticulum and syringomyelia resolved after 3 months, revision surgery became necessary when the shunt catheter was displaced, causing neurological deterioration (7). This underscores both the potential effectiveness and the technical challenges of placing and securing shunts in confined spaces.

A more comprehensive overview of each technique is provided in Table 1.

**Table 1:** Comprehensive comparison of the surgical techniques described for the management of Chiari-like malformation and syringomyelia in dogs, including indications, advantages, disadvantages, and reported outcomes.

Surgical technique	Number of dogs	Reported success	Long-term medication post-operative	Long-term medication post-operative MRI
FMD without cranioplasty	21 CKCS	−81% showed clinical improvement after surgery. - 5% required revision surgery.	19% dogs needed medication for flare-ups.	In 48% at a mean of 6.2 months (2–13 months). - Three dogs had no pre-operative SM and remained SM-free. In 7 dogs, the syrinx appeared unchanged post-operatively.
FMD with durotomy without cranioplasty	15 CKCS	−80% clinical improvement, but only 1 (12.5%) had resolution of clinical sings. - 20% remained unchanged.	47% eventually deteriorated (0.2 and 2.3 years post-surgery), needing ongoing medication.	In the one available (40%), SM persisted.
FMD with occipital cranioplasty (titanium prothesis)	8	3/5 had a 30–50% syrinx decrease, 1/5 had a 12% increase, and 1/5 had complete resolution.	63% required long-term medication (low-dose prednisolone)	MRI at 6–20 months post-operative. - 3 dogs had a 30–50% decrease in syrinx - 1 had a 12% increase in syrinx size. - 1 had complete resolution syrinx.
Syringo-subarachnoid shunting	11 CKCS and Yorkies	- Neurological improvement in 81%. - 63% survival. - 9% required a second procedure.	All continued furosemide (except 2 with side effects)	Not available.
Syringo-subarachnoid shunting (case report)	1 CKCS	Significant reduction in neuropathic pain, but intermittent episodes remained	Unknown.	Not available.
Syringo-pleural shunting	1 Pug	Resolution of syringomyelia after 3 months but required revision surgery.	Yes, the specific medications are not specified	No available.

Complications in dogs with ventriculoperitoneal shunts include infection, undershunting caused by dislocation, kinking of the catheter, leakage, valve breakage, skin necrosis, craniostenosis, microcephaly, aqueduct stenosis or obstruction, headaches, or overshunting resulting in ventricular collapse and subdural hematoma and hygroma. Overshunting may lead to the development of subdural hematomas and hygromas (19, 20). In one study, shunt catheter obstruction was the most common cause for shunt malfunction (21). Infection is the second most usual cause of malfunction with a reported rate of approximately 8–15% among patients undergoing ventriculoperitoneal shunt placement. In three dogs, cystic hygroma was observed after ventriculoperitoneal shunt placement on the repeated MRI 1 month later (22). Pneumocephalus has not been reported in dogs post-operatively with a ventriculoperitoneal shunt; however, it has been described in humans (23). In this case, it was likely caused by the repositioning of the shunting tube. A high-pressure valve was chosen due to the expected slow flow and low pressure to be associated with the lack of hydrocephaly and lack of signs of raised intracranial pressure.

A larger number of cases would be necessary to best determine the long-term outcome of this surgical treatment. However, such numbers might be difficult to obtain given that surgical management is more invasive, with a vast repertoire of complications, and SM is often successfully managed medically.

## 5 Conclusion

This report describes successful management of CM-associated syringomyelia and ventriculomegaly using a ventriculoperitoneal shunt in a young Cavalier King Charles Spaniel. Clinical signs resolved, MRI demonstrated progressive reduction of both the syrinx and ventriculomegaly, and the dog remained free of medication 2 years after surgery. Although additional cases are required before firm recommendations can be made, this outcome suggests that VPS may be a valuable treatment option in selected patients with advanced CM/SM and ventriculomegaly.

## Data Availability

The original contributions presented in the study are included in the article/supplementary material, further inquiries can be directed to the corresponding author.
